# Reliability of acoustic tomography and ground‐penetrating radar for tree decay detection

**DOI:** 10.1002/aps3.1187

**Published:** 2018-10-23

**Authors:** Xi Wu, Guanghui Li, Zhi Jiao, Xiping Wang

**Affiliations:** ^1^ School of IoT Engineering Jiangnan University No. 1800 Lihu Road, Binhu District Wuxi Jiangsu 214122 People's Republic of China; ^2^ USDA Forest Service Forest Products Laboratory 1 Gifford Pinchot Drive Madison Wisconsin 53726‐2398 USA

**Keywords:** image processing, MATLAB, nondestructive testing, PiCUS 3 Sonic Tomograph, resistance micro‐drilling, Tree Radar Unit (TRU) System

## Abstract

**Premise of the Study:**

Various nondestructive testing technologies have been used for detecting and visualizing internal defects in urban trees. The results obtained by using different nondestructive testing tools can be interpreted in different ways, which may result in inaccurate assessment of the true condition of the inspected trees. The main objective of this study was to evaluate the reliability of acoustic tomography and ground‐penetrating radar (GPR) technology for detecting internal decay in a number of different tree species.

**Methods:**

One hundred and forty‐seven individual trees of 33 species were inspected at a historic park in Yangzhou, Jiangsu Province, China, using a combination of visual inspection, acoustic tomography, GPR scanning, and resistance micro‐drilling methods. Special image processing procedures were developed to analyze the acoustic and radar images and to estimate the proportion of compromised wood.

**Results:**

The acoustic tomography tests revealed 10 defective trees with acoustic shadows suggesting compromised wood in more than 10% of the cross‐section area. The actual nature of these defects on living trees can be ascertained by conducting resistance micro‐drilling at selected paths. The Tree Radar Unit (TRU) System produced 85% false positive predictions and thus was not successful in visualizing the true physical conditions of the trees.

**Conclusions:**

Acoustic tomography can successfully detect trees with internal decay and cavities. A combination of visual inspection, acoustic tomography, and resistance micro‐drilling is an effective approach to detect, measure, and visualize internal defects on a diversity of tree species. The image processing procedures we developed make possible the quantitative analysis of compromised wood and could improve the science‐based tree risk assessment process. In contrast, the TRU System presented challenges in field applications, particularly on trees with small diameters and irregularly shaped trunks. The images obtained in this study using the TRU system were largely inaccurate and not reliable for tree inspection.

Trees are valuable assets within an urban community, providing ecological, aesthetic, social, and economic benefits. The structural failure and mortality of urban trees caused by internal rot, insect attack, and extreme wind loads can cause significant property damage and pose a threat to public safety. In tree stability analysis, arborists use both biological and engineering principles to rate a tree's internal soundness and make reasonable predictions of potential for structural failure (Wang and Allison, 2008). In practice, arborists and tree managers are often challenged by internal decay and other structural defects that are hidden from view and thus have long sought noninvasive and cost‐effective tools to detect, measure, and visualize internal defects in trees (Johnstone et al., 2007; Wang and Allison, 2008; Allison and Wang, 2015; Gilbert et al., 2016).

In the past two decades, nondestructive testing and evaluation of urban trees for decay detection has drawn attention from the research community around the world (Arciniegas et al., 2014). Tree care professionals have also shown an increasing interest in applying more rapid and precise diagnostic techniques to detect decay and other types of structural defects in trees (Allison et al., 2008; Koeser et al., 2017). Commercial noninvasive tools using acoustic waves (single path and multiple paths), electromagnetic waves (ground‐penetrating radar [GPR]), and mechanical micro‐drillings are now available for field application (Allison and Wang, 2015).

The principles of the stress wave timing method for decay detection in standing timber have been provided by Wang et al. (2004). In field applications, this nondestructive testing procedure is also referred to as single‐path stress wave timing measurement. The stress wave velocity obtained through measuring stress wave transmission time and tree diameter has been found to be directly related to the physical condition of the tree. In general terms, stress waves travel faster in defect‐free trees. Areas of rot, cavities, or internal cracks will require a longer time for the stress wave to travel through the material (Wang et al., 2004). The success of using the single‐path stress wave timing method to detect internal decay in live trees has been reported in many papers (McCracken, 1985; Mattheck and Bethge, 1993; Yamamoto et al., 1998). However, this method is limited in its ability to detect moderate to severe internal defects in trees, as it is not sufficiently sensitive to detect internal decays at an early stage or of small size (Li et al., 2016).

Acoustic tomography technology uses multiple sensors (usually eight to 32) to measure stress wave transmission times in multiple directions. Computer projection software then uses the acoustic wave data matrix to create an image (tomogram) of the apparent acoustic wave velocity for the cross section of the tree. Although the acoustic tomograms obtained from urban trees with central rot have shown good agreement with the true physical condition for the cross section, an acoustic tomogram is not a spatially precise map of the internal structure, instead it represents the wood's acoustic characteristics as determined by the perceived velocities (Wang et al., 2009; Allison and Wang, 2015; Lin and Yang, 2015). Consequently, the interpretation of the acoustic tomogram is not a straightforward process and often requires experience and further verification by other methods such as resistance micro‐drilling. Field evaluation of an individual tree's condition solely based on acoustic tomograms can sometimes lead to false diagnosis (Wang et al., 2009; Kloiber et al., 2016).

The potential of using GPR technology to inspect urban trees and discern internal defects in tree trunks has also been explored by some researchers. GPR is a geophysical method that uses an antenna to propagate short bursts of electromagnetic energy into solid materials and measures the two‐way travel time and amplitude of reflected signals. As radar wave pulses propagate through a boundary of two solid mediums with different properties, the electromagnetic waves reflect and refract according to the law of physics. This technology has been in use for more than 30 years and is commonly used to locate underground engineering and environmental targets without the need for excavation. Recent studies indicate that internal decay in trees, which results in changes in wood density and moisture contents, can be detected by GPR scanning in single‐reflection mode. In an exploratory study, Nicolotti et al. (2003) employed high‐frequency radar (1.5‐GHz center frequency) to identify areas of decay in a plane tree (*Platanus hybrid* Brot.) in an urban setting. By advancing a single antenna around the circumference of the tree, they were able to acquire data in single‐reflection mode. The linear two‐dimensional data were transformed into polar coordinates to display a radar image for an easier and more realistic comparison with the tree sections. The authors reported that the area of decay exhibited increased dielectric properties, and there was a good agreement between radar assessment of decay and destructive sampling via physical means. Butnor et al. (2009) also applied GPR technology (900‐MHz center frequency) to nondestructively estimate the decay volumes in coniferous trees through circumference GPR scanning at multiple elevations (expert tree climbers were employed to scan 10–15 different elevations per tree). They reported that near‐surface decay, air‐filled voids, and desiccated boles exhibited unique electromagnetic signatures that can be used to detect structural defects on trees. The percentage of air‐filled cavities on those trees was estimated with reasonably good agreement with the destructive sampling (Butnor et al., 2009). Jezova et al. (2016) investigated the particularities of tree trunk radar images, considering the circumferential data acquisition geometry as a function of the radar configuration and trunk section structures. The experimental results showed the internal inhomogeneity; this information will be useful for future tomographic reconstruction.

The greatest challenges in using GPR to inspect urban trees are the difficulty in coupling the antenna to the curved, often irregular bark surface of the tree trunk and the interpretation of complex data (Schad et al., 1996; Nicolotti et al., 2003; Butnor et al., 2009). It is important that the antenna maintain good contact with the bark, as large air gaps or operator “wobbles” degrade the quality of the scan. In addition, interpretation may be complicated by differences between tree species, trunk diameters, moisture gradients related to heartwood development, and environmental conditions. More research is needed to outline the full range of detectable defects and thus advance the field applications of GPR technology.

Resistance micro‐drilling is a widely used inspection tool for both tree decay detection and timber structure condition assessment (Rinn, 1990, 2012; Ceraldi et al., 2001; Allison and Wang, 2015). Resistance micro‐drilling uses a thin, rotating needle (tip diameter 3 mm, shaft diameter 1.5 mm) to drill through wood with a constant speed, across the grain. The relative drilling resistance can be continuously displayed and recorded in real time. A prolonged low resistance in a resistance profile typically indicates decay, cavities, or large internal cracks. Many field tests have proved that the damage caused by drilling with a thin needle is minimal. In practice, resistance micro‐drilling is usually used as a confirmation test to locate any type of internal defects in suspect trees (Wang and Allison, 2008).

In this paper, we report the results of an inspection project using a combination of acoustic tomography, GPR, and resistance micro‐drilling methods to assess the conditions of 147 living trees of 33 species at a historic city park in Yangzhou, Jiangsu Province, China. The main objectives of this study were to evaluate the reliability of acoustic tomography and GPR imaging technologies in assessing a diversity of tree species with different types of internal defects and to provide a science‐based report of tree conditions to the park managers for future monitoring and conservation actions.

## MATERIALS AND METHODS

A field tree inspection project was conducted at Slender West Lake, a historic city park in Yangzhou, Jiangsu Province, China, from 30 August to 6 September 2016. Yangzhou is a historic city dating back to the fifth century BC. Located in Yangzhou's northern suburb, Slender West Lake is a fine example of a traditional Chinese lakeside garden with many historic trees. In this project, a total of 157 live trees of 33 species were surveyed (Table 1); the trees ranged in age from 90 to 300 years old. Among the surveyed trees, 147 trees were inspected using a combination of visual inspection, acoustic tomography, GPR, and resistance micro‐drilling. Ten trees were only inspected visually because of accessibility issues. The tree code assigned to each tree by the park manager was used as the tree ID in the inspection.

**Table 1:** Thirty‐three tree species surveyed and tested at Slender West Lake, Yangzhou, China

Species	No. of trees
Chinese juniper (*Juniperus chinensis* L.)	30
Pecan (*Carya illinoinensis* (Wangenh.) K. Koch)	19
Ginkgo (*Ginkgo biloba* L.)	16
Sweet olive (*Osmanthus fragrans* (Thunb.) Lour.)	15
*Juniperus chinensis* ‘Kaizuka’ (*Sabina chinensis* (L.) Antoine)	7
Chinese boxwood (*Buxus sinica* (Rehder & E. H. Wilson) M. Cheng)	6
Mulberry (*Morus alba* L.)	6
Japanese maple (*Acer palmatum* Thunb.)	6
Chinese arbor‐vitae (*Platycladus orientalis* (L.) Franco)	6
Magnolia (*Magnolia grandiflora* L.)	5
Chinese hackberry (*Celtis sinensis* Pers.)	5
Mono maple (*Acer mono* Maxim.)	4
Crape myrtle (*Lagerstroemia indica* L.)	4
Maple poplar (*Pterocarya stenoptera* C. DC.)	3
Chinese tallow tree (*Sapium sebiferum* (L.) Dum. Cours.)	3
Cedar (*Cedrus deodara* (Roxb.) G. Don)	2
Japanese cedar (*Cryptomeria japonica* (L. f.) D. Don)	2
*Cryptomeria* (*Cryptomeria japonica* cv. *‘*Araucarioides’)	2
Chinese cedar (*Cryptomeria japonica* cv. ‘Yua’)	2
Water elm (*Zelkova serrata* (Thunb.) Makino)	1
Trident maple (*Acer buergerianum* Miq.)	1
Kingwood (*Dalbergia hupeana* Hance)	1
White pine (*Pinus bungeana* Zucc. ex Endl.)	1
Elm (*Ulmus parvifolia* Jacq.)	1
Chinese soapberry (*Sapindus* L.)	1
Javanese bishopwood (*Bischofia javanica* Blume)	1
Chinese mahogany (*Toona sinensis* (A. Juss.) Roem.)	1
Magnolia tree (*Michelia alba* DC.)	1
White flower catalpa (*Catalpa speciosa* Teas)	1
Chinese walnut (*Juglans cathayensis* Dode)	1
Camphor tree (*Cinnamomum camphora* (L.) J. Presl)	1
Chinese tulip tree (*Liriodendron chinense* (Hemsl.) Sarg.)	1
Turnjujube (*Hovenia acerba* Lindl.)	1

A visual inspection was first conducted on each tree to document visual signs of structural instability (e.g., physical damages, exterior cavities, fungal growth) with photographic documentation and to determine the locations of tree trunks requiring further nondestructive testing. A combination of acoustic tomography, GPR, and resistance micro‐drilling was used for nondestructive testing at selected elevations along the tree trunk. The general procedures of nondestructive testing are as follows:

1. Conduct acoustic tomography measurements at the selected elevations on each tree trunk using a PiCUS 3 Sonic Tomograph tool (Argus Electronic GmbH, Rostock, Germany). The measurement was performed at a low elevation (30 to 50 cm aboveground, avoiding buttresses) where internal decay is most likely to occur. If a major defect was diagnosed from the resulting tomogram at a low elevation, one or two additional acoustic tomograph measurements were conducted 50 to 100 cm above the initial elevation. The majority of the trees were tested using a standard protocol with 12 sensors. Some smaller trees (such as *Buxus sinica* (Rehder & E. H. Wilson) M. Cheng) less than 20 cm in diameter were tested using six to 10 sensors. In principle, as the number of sensors around the circumference of a trunk increases, the image quality and accuracy improves. However, increasing the number of sensors also increases the measurement time. Therefore, using fewer sensors has a time advantage if the resulting tomograms still provide satisfactory resolution.
2. Conduct GPR testing using the Tree Radar Unit (TRU) System (TRU‐900; Tree Radar Inc., Silver Spring, Maryland, USA) with a 900‐MHz radar antenna. The GPR scanning was performed at the same elevations where acoustic tomograph measurements were conducted and began with the same starting point as the acoustic tomograph measurements (sensor no. 1). During the scanning, the field data acquisition computer automatically generates a radar waveform for every 5 mm of the circumference traversed. The reflected radar waveforms were continuously collected while the operator moved the radar antenna around the trunk's cross section with the antenna in contact with the bark. A complete 360‐degree pass around the tree trunk produces hundreds of waveforms that are displayed on the field computer as a radargram. One of the disadvantages of the TRU‐900 unit is the large size of the radar antenna (18.5 × 32.5 cm), which is not suitable for scanning small trees (<20 cm diameter). In this project, we only scanned 60 trees using the TRU‐900 unit. GPR scanning began at the initally tested low elevation from the acoustic tomograph measurements. When a major defect was diagnosed from the resulting radargram at the low elevation, one or two additional GPR scans were conducted at higher elevations (as defined above for the acoustic tomography elevations). The TRU System scanning of 60 trees resulted in a total of 109 images of predicted internal cross‐sectional views (called “virtual saw cut”).
3. After acoustic tomography tests and GPR scanning, a resistance micro‐drilling tool (RESISTOGRAPH 4452‐P; RinnTech Inc., Heidelberg, Germany) was used to verify some of the defect areas indicated by acoustic tomograph or GPR images. To limit the number of drillings, resistance micro‐drilling was performed on 11 selected paths of the suspect trees. The drilling paths were selected to enter the area of trunk cross section displayed in the tomograph as possible decay. The goal was to determine if the tomograph display represented an area of hollow, decay, or a crack‐induced acoustic shadow.

### Data processing

#### Construction of acoustic tomograms

The wave transmission time data obtained from acoustic tomograph measurement at each tree elevation were used to generate the two‐dimensional acoustic velocity distribution map (tomogram) using the PiCUS 3 software. Acoustic tomograms were constructed in a predesignated color scheme: brown (dark brown/light brown), green, and violet/blue/white, with dark brown representing the high acoustic velocity (high density and sound wood), violet/blue/white representing areas of low acoustic velocity (low density and unsound wood), and green representing the transition zone (barrier wall) between deteriorated and sound wood. The color scale is expanded between 100% and the lowest velocity.

To eliminate false indication of decay near the perimeter between sensors (a common problem in PiCUS tomograms), we applied the “cogwheel correction filter” to generate the final tomogram. This procedure does not affect accurate visualization in the absence of the cogwheel artifact and greatly reduces false positives from a known problem, as described in the PiCUS manual (Argus Electronic, 2013).

#### Construction of TRU radar image (virtual saw cut)

The GPR scan data obtained from selected elevations were analyzed using the TreeWin‐Trunks analysis module (Tree Radar Inc.), a post‐processing and data analysis software. The analysis of the recorded radargram data resulted in a cross‐section image (hereafter referred to as radar image) that represents the internal trunk structure for each elevation scanned. Unlike the multi‐colored acoustic tomograms, the image generated by the TRU software contains only two colors: an orange area representing compromised wood (decay or cavity) and a light brown area indicating solid wood.

#### Quantification of compromised wood in tree trunks

Both the acoustic tomograms and radar images use color schemes to represent the potentially compromised wood in tree trunks, but no quantitative data were provided for the proportion of the defect areas. To assess the accuracy and reliability of these two technologies, we developed special image processing procedures to estimate the proportion of the compromised wood represented by color areas in the tomograms and radar images. All image processing operations were performed in the MATLAB environment. The acquired images were subjected to a sequence of image pre‐ and post‐processing operations to determine the proportion of compromised wood.

**Figure 1:**
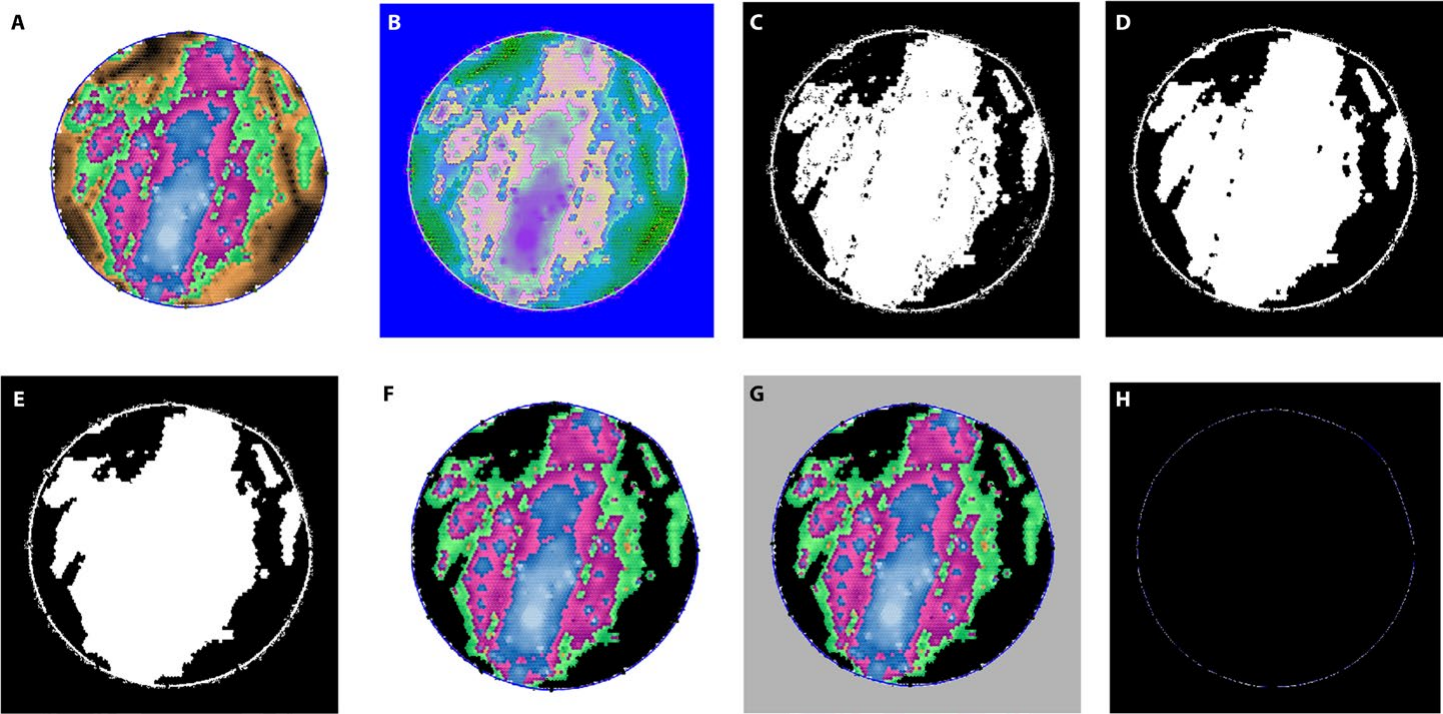
PiCUS image processing steps: (A) original image without axis information, (B) image under HSV color model, (C) original image transformed to a binary image (black and white), (D) binary image with some erroneous areas deleted, (E) binary image without small erroneous areas, (F) original image superimposed over the white region in the binary image, (G) background color changed to gray, (H) edge of the tree trunk (blue circle) isolated.

Different procedures were used to process PiCUS tomograms and TRU radar images. The radar images were processed in their original RGB (red, green, and blue) model. The proportion of the compromised wood was determined by counting the pixels in the orange areas and dividing that number by the number of pixels in the entire cross section. The PiCUS acoustic tomograms contain multiple colors and are therefore more complex to deal with. To facilitate color processing and recognition, the image segmentation of the PiCUS tomogram was performed under the HSV (hue, saturation, and value) model. The HSV color model comes from the human visual system, and describes the color by hue, saturation, and value. It effectively identifies color divisions. The image processing procedures are illustrated in Fig. 1 and include the following steps: (1) Read the original image (Fig. 1A) without axis information. (2) Transform the image to a 580 × 580 × 3 matrix using the HSV color model (Fig. 1B). (3) Using the pixel information in the matrix, transform the image to a binary image (pixel value is 1 [white] or 0 [black]) as shown in Fig. 1C. (4) Remove undesired pixels (i.e., spurious and isolated pixels) from the resulting binary image (Fig. 1D, E). This is realized by using a binary morphological operator available in the “*BW2 = bwareaopen (BW, P, conn)*” function in MATLAB. (5) Superimpose the original colored image over the white region in the binary image, and cut off the black background outside the cross section. After this step, brown regions on the cross section are clearly shown in black (Fig. 1F). (6) Extract the edge of the image by traversing all pixel points outside of the blue boundary and change these to gray color (Fig. 1G). The edge of the image is shown in Fig. 1H as a blue circle. The numbers of pixels in colored areas and solid regions were then used to calculate the proportion of the compromised wood in the cross sections.

## RESULTS AND DISCUSSION

The majority of the trees we surveyed at Slender West Lake were found to be in good condition. Among the 147 trees that were nondestructively tested, 10 trees were identified to have internal decay, cavities, or internal cracks. Table 2 summarizes the evaluation results of the defective trees identified using the PiCUS and TRU tools and confirmed through resistance micro‐drilling and visual inspection.

**Table 2:** Defective trees identified using PiCUS and TRU tools and confirmed through resistance drilling and visual inspection

Tree no.	Species	Age (y)	Elevation (cm)^a^	Trunk diameter (cm)	Compromised wood (%)		Visual observation	Resistance drilling path (Confirmation)
					PiCUS	TRU		
14	*Hovenia acerba*	NA	125	43.9	23.5	NT	Open cavity, internal rot	NT
30	*Juniperus chinensis*	130	62	46.5	64.4	NT	Seams	1–7 (+), 2–9 (+), 4–10 (+)
64	*Cryptomeria japonica* cv. ‘Yua’	120	30	31.8	25.8	NT	Seams, bark decay	NT
71	*Juniperus chinensis*	130	45	49.7	19.8	NT	Open cavity	10–6 (+), 10–5 (+), 7–12 (+)
76	*Platycladus orientalis*	120	160	29.6	11.3	10.4	Good	NT
90	*Acer palmatum*	90	25	34.4	37.9	NT	Open cavity, internal rot	3–8 (+), 4–7 (+), 5–10 (+)
100	*Morus alba*	120	115	79.0	41.4	17.3	Severe decay	9–1 (+), 2–11 (+)
102	*Morus alba*	120	115	79.6	71.8	NT	Severe decay, open cavity	NT
104	*Morus alba*	120	137	77.4	42.1	20.1	Severe decay	NT
109	*Celtis sinensis*	90	120	60.5	21.9	37.3	Good condition	NT

Note

+ = positive confirmation; NA = not available; NT = not tested.

a Elevation on the tree trunk at which PiCUS and TRU testing was performed.

**Figure 2:**
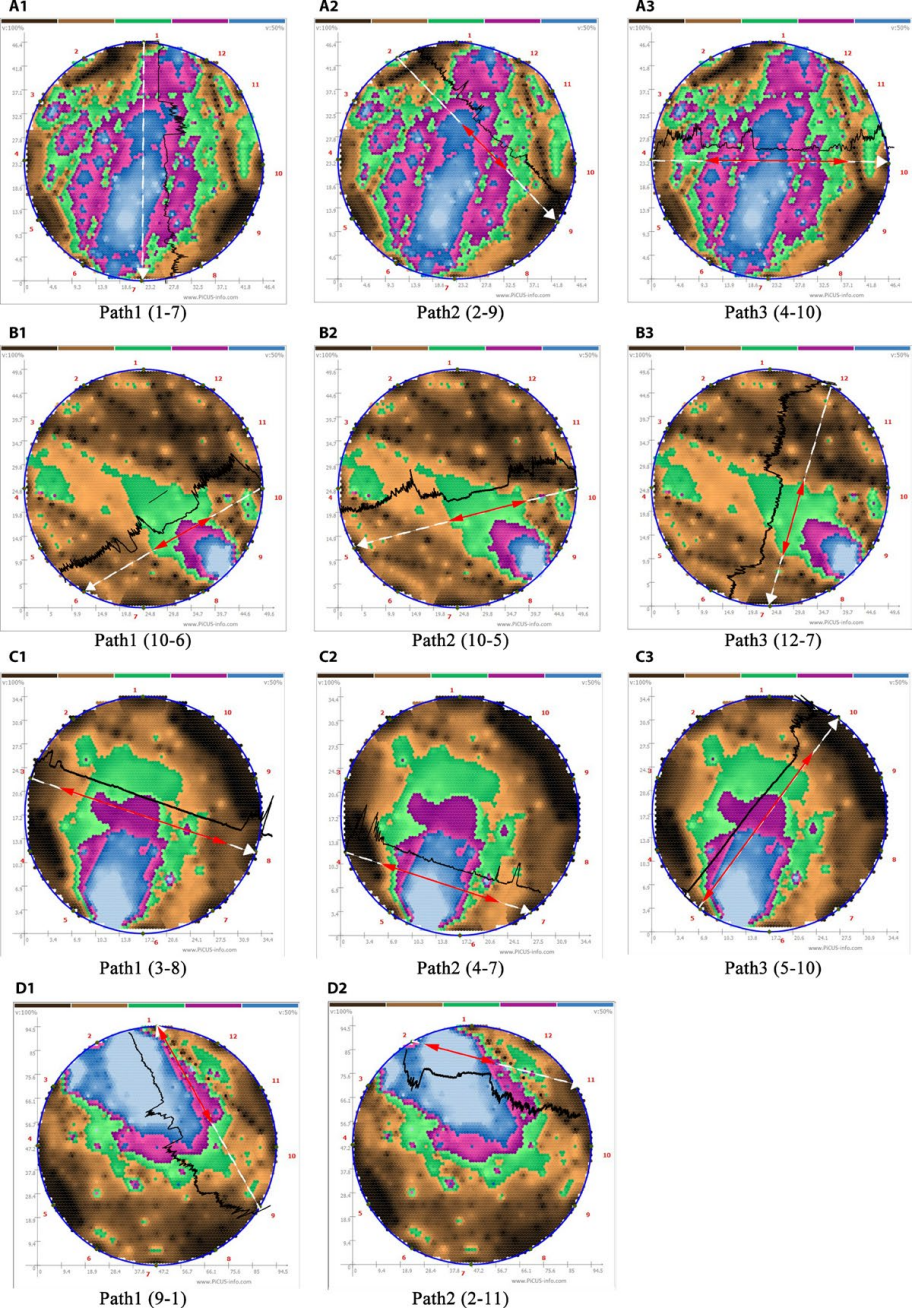
The PiCUS tomogram superimposed with the RESISTOGRAPH profile in selected drilling paths. A1–A3 = tree no. 30, B1–B3 = tree no. 71, C1–C3 = tree no. 90, D1–D2 = tree no. 100. The white dashed arrows indicate the drilling path and drilling direction, the red arrows show the depth of the decay region, and the black lines represent the superimposed resistance profiles.

The acoustic tomography tests revealed 10 trees with acoustic shadows (compromised wood) in more than 10% of the cross‐section area (Table 2). Among those 10 trees, five trees (no. 30, 90, 100, 102, and 104) were found to potentially have extensive internal decay or cavities exceeding one third of the cross section (37.9–71.8%). Further testing using the RESISTOGRAPH tool on the selected paths (trees no. 30, 71, 90, and 100) and careful visual examination confirmed the presence of decay, cavities, and large internal cracks on these trees. A precise quantification of defect location and size on selected trees was achieved by superimposing the resistance profiles with the acoustic tomograms as shown in Fig. 2.

In each of the images in Fig. 2, white dashed arrows indicate the drilling path on the tree trunks, black lines represent the superimposed resistance profiles, and red arrows show the depth of internal decay or cavity. In the case of tree no. 30 (Fig. 2A), the compromised wood area accounted for 64.4% of the trunk cross section. Resistance profiles matched the size of the compromised wood in path 3 (4–10), but not in path 1 (1–7) or path 2 (2–9). This indicates that the defect area is likely a combination of internal rot and a large lateral crack extending to the edge of the trunk. In this case, it is difficult to estimate the exact size of internal decay, but the presence of one or multiple large lateral cracks has a dominating effect on the tomogram. In trees no. 71 (Fig. 2B), 90 (Fig. 2C), and 100 (Fig. 2D), the resistance profiles matched the area of the compromised wood in almost all of the drilling paths, indicating internal decay and cavities are the main defects in these trees.

**Figure 3:**
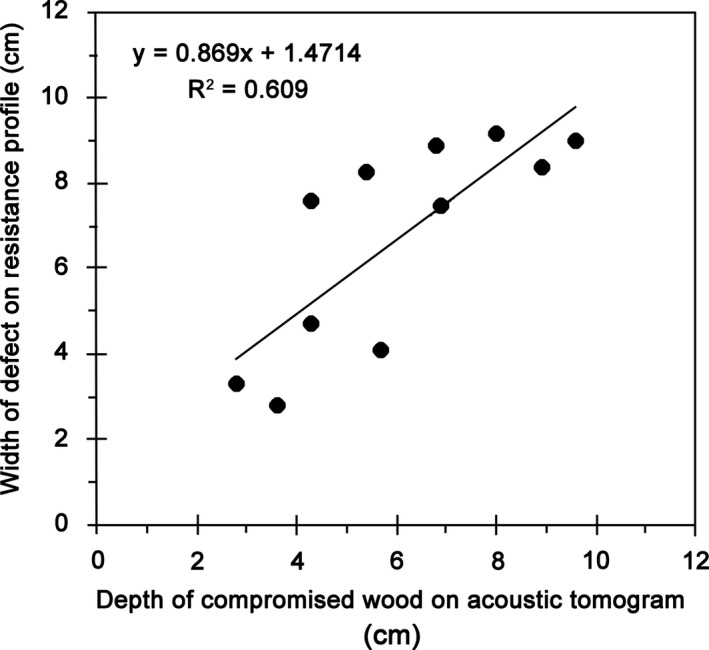
Relationship between the depth of the compromised wood as indicated by the acoustic tomograms and the defect length detected on the resistance profiles.

At each drilling path, the depth of the compromised wood on the acoustic tomogram was determined and compared against the width of decay detected on the resistance profiles (Fig. 3). Despite some mismatches, the depth of the compromised wood shown on the acoustic tomograms had a good linear relationship with the defect width determined by the resistance drilling test (*R* ^2^ = 0.61), indicating that acoustic tomography is a reliable inspection tool.

Trees no. 102 and 104 both contain open cavities and compartmentalized decay that can be visually observed. The significant proportion of the compromised wood on these five trees (no. 30, 90, 100, 102, and 104) suggest that they are in high risk of failure and special precaution measures should be taken.

**Figure 4:**
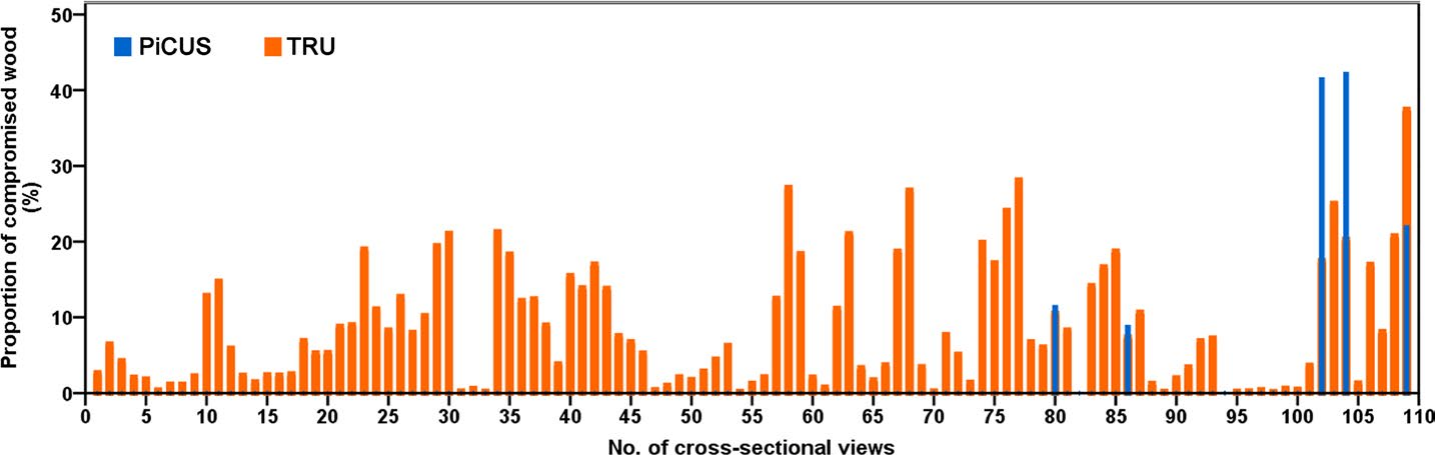
Percentage of compromised wood predicted by TRU and PiCUS (109 elevations measured on 60 trees).

The TRU scan data from 60 trees resulted in 109 radar images, one for each elevation scanned on each tree. The results of quantitative analysis of the radar images are shown in Fig. 4, along with the analysis results of the PiCUS tomograms. Among all of the elevations tested, radar images detected 37 elevations with areas of compromised wood of more than 10% of the cross section and 11 elevations with areas of compromised wood of more than 20% of the cross section. It was found that TRU radar images resulted in false positive predictions on 51 trees and 99 elevations, where both visual inspection and acoustic tomography testing indicated sound and healthy trunks but radar images showed compromised wood.

**Figure 5:**
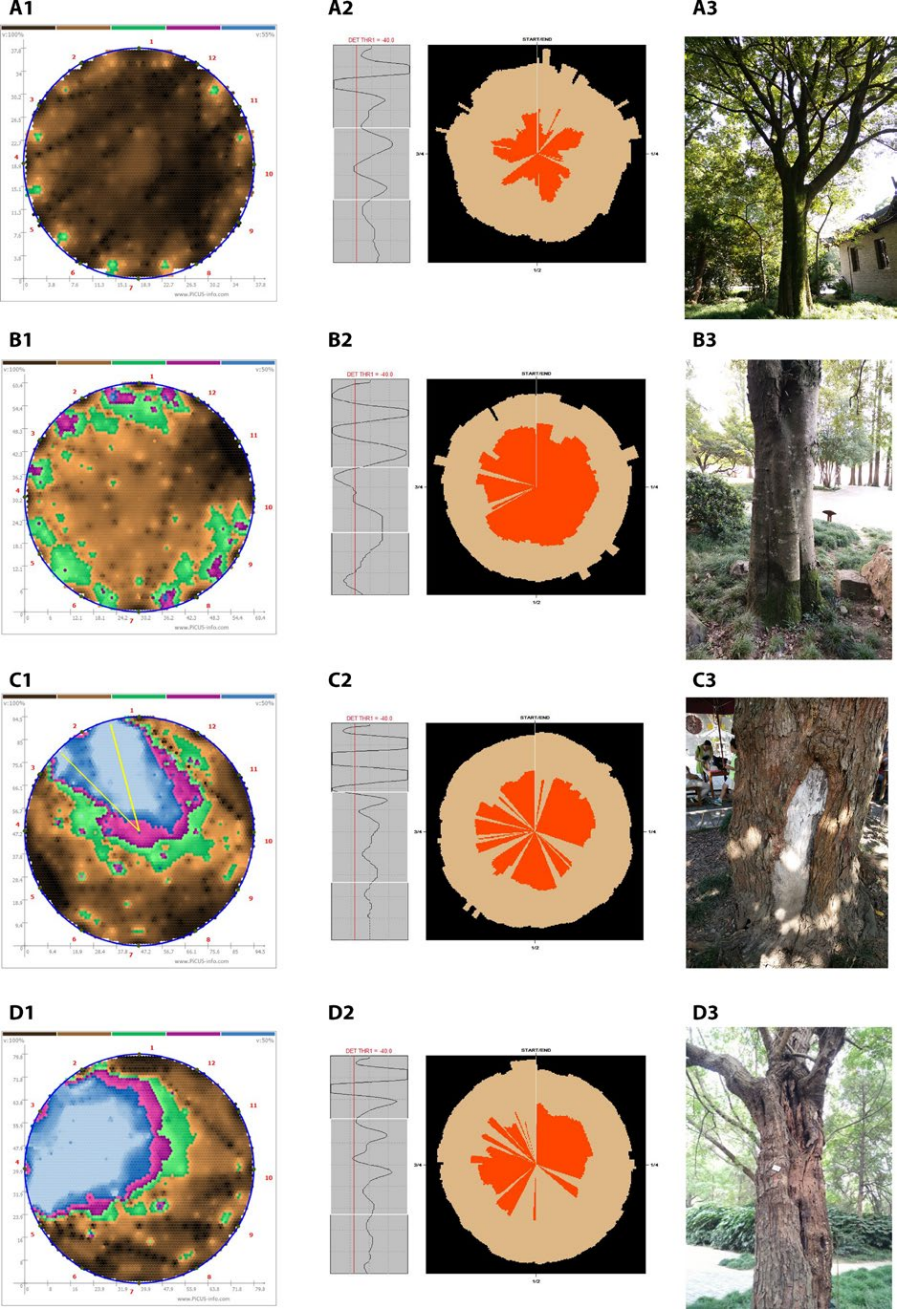
Comparison of the TRU radar image and the corresponding PiCUS tomogram for a healthy tree (A) and for defective trees (B–D). A1–A3 = tree no. 78, B1–B3 = tree no. 109, C1–C3 = tree no. 100, D1–D3 = tree no. 104. The proportion of compromised wood for tree no. 78: 14% (TRU) vs. 0% (PiCUS); tree no. 109: 37.3% (TRU) vs. 21.9% (PiCUS); tree no. 100: 17.3% (TRU) vs. 41.4% (PiCUS); and tree no. 104: 20.1% (TRU) vs. 42.1% (PiCUS).

For four selected trees of different conditions, the TRU radar images and PiCUS tomograms were compared (Fig. 5). For tree no. 78, which was indicated as being sound and healthy by the PiCUS tomogram and visual inspection, the radar image indicated a center decay area of 14% (i.e., a false positive). Fifty‐one out of the 60 trees scanned by TRU fell into this category, representing 85% false positive predictions. For trees no. 109, 100, and 104, radar images correctly detected the presence of internal decay and cavities, but the location and size of the defect do not match the corresponding PiCUS tomograms as shown in Figures 5B–D.

The results of TRU images from this study indicate that GPR scanning presents challenges for accurately inspecting tree trunks of many species. The false positive predictions are partially due to (1) the rugged bark surface and irregular shapes of many tree trunks, which can cause “wobbling” during scanning; (2) oversized radar antenna (18.5 × 32.5 cm) that are not suited to smaller trees and that created large uneven air gaps across the width of the antenna; and (3) relatively low center frequency of the antenna (900 MHz). Even for the true positive predictions on some defective trees, the radar images produced by the TreeWin‐Trunks analysis module showed large discrepancies with the PiCUS tomograms, an indication of possible flaws that may exist in the algorithm for post‐radargram processing.

## CONCLUSIONS

The majority of the trees we surveyed and nondestructively tested at Slender West Lake were in good condition, which is the result of the high levels of tree maintenance practices by the park managers and tree care professionals. Ten defective trees were identified, of which five were rated as high risk, and special treatment and safety actions were recommended.

The results of this study demonstrated the effectiveness of using a combination of visual inspection, acoustic tomography, and resistance micro‐drilling to detect, measure, and visualize internal defects on a diversity of tree species. The image processing procedures we developed in the MATLAB environment make it possible to conduct quantitative analysis on compromised wood, which could facilitate the implementation of acoustic tomography technology and improve the science‐based tree risk assessment process. The TRU System, on the other hand, presented challenges in field applications and especially on trees of small diameter and with irregularly shaped trunks. The tree radar images obtained in this study were largely inaccurate and not reliable for tree inspection purpose.
